# An innovative approach to developing practice informed evidence in the absence of RCTs for a Family-led peer to peer support program

**DOI:** 10.23889/ijpds.v9i5.1681

**Published:** 2024-09-18

**Authors:** Alicia Montgomerie, Vita Maiorano, Danielle Abbott, John Lynch, Rhiannon Pilkington

**Affiliations:** 1 School of Public Health, The University of Adelaide; 2 The Australian Centre for Social Innovation; 3 School of Public Health, The University of Adelaide; 4 School of Population Health Sciences, University of Bristol

## Objective

To illustrate how whole-population linked data can be used to understand a system perspective of client complexity, and build robust evidence of Family by Family program impact.

## Methods

Family by Family program (the program) participant data were linked into the Better Evidence Better Outcomes Linked Data (BEBOLD) Platform. BEBOLD is a whole-of-population linked de-identified administrative data platform for all South Australian children born 1991 onwards (n~500,000), as well as their parents including data spanning health, education, and social services.

We descriptively analysed parental child protection history, emergency department presentations, hospitalisations, homelessness and justice system contact in the 24 months prior to and post program commencement. We emulate a trial using the ‘target trial’ causal inference framework to evaluate the program effect on a range of child outcomes using targeted maximum likelihood with a set of over 20 confounders.

## Results

There were 361 families and 841 children in the program included in analysis. Selected results follow: Prior to the program, 35.8% of children were in a family where at least one parent had their own child protection history and 8% had a parent who experienced out-of-home care. Nearly 40% of children had at least one parent with a mental health related emergency department and/or hospitalisation, while 22% of children were in a family with specialist homelessness service contact. Program impact results will be presented at the conference.

## Conclusion

This research-practice partnership illustrates how bringing together program and linked-administrative data generates new evidence about client complexity and program impact.

